# Guillain-Barré Syndrome-Like Polyneuropathy Associated with Immune Checkpoint Inhibitors: A Systematic Review of 33 Cases

**DOI:** 10.1155/2021/9800488

**Published:** 2021-08-19

**Authors:** Yan Li, Xiuchun Zhang, Chuansheng Zhao

**Affiliations:** Department of Neurology, The First Hospital of China Medical University, Shenyang 110001, China

## Abstract

Immune checkpoint inhibitors (ICIs) have been increasingly used in the treatment of various types of tumors with favorable results. But these treatments also led to a variety of immune-related adverse events (irAEs). Neurological irAEs such as Guillain-Barré Syndrome are rare and may have serious consequences once they occur. A systematic literature search was performed in PubMed and Embase for all case reports of GBS associated with ICIs published in English reporting on human beings from 1990 up to date. A total of 30 case reports (total patients = 33) were used for final analysis. The included cases were from 11 countries, covering 10 tumor types, with melanoma accounting for the largest number. The mean age was 62.2 ± 11.1 years old, and males were dominant (male: 26 and female: 7). The median time of initial symptoms was 8.2 weeks after the 1^st^ dose of ICIs. The most common manifestations of GBS associated with ICIs were weakness, hyporeflexia or areflexia, and paresthesia in order. The GBS subtypes suggested by electrophysiological results were acute inflammatory demyelinating polyneuropathy (AIDP), acute motor axonal neuropathy (AMAN), and Miller Fisher syndrome (MFS). The protein level of CSF in patients with GBS related to ICIs was 180.68 ± 152.51 mg/dl. Immediate termination of ICIs followed by intravenous immunoglobulin was the preferred treatment option. 72.7% of patients recovered or had residual mild dysfunction after treatment. Elderly male patients with melanoma were most likely to develop ICI-related GBS. The specific neurological symptoms, CSF analysis, and electrophysiological examination were important means of diagnosis.

## 1. Introduction

In the last decade, with a better understanding of the factors that promote or inhibit T cell response, great progress has been made on tumor immunotherapy. Immune checkpoint inhibitors (ICIs) have become a powerful clinical strategy for treating cancer, including an antibody targeting cytotoxic T lymphocyte-associated antigen-4 (CTLA-4, e.g., ipilimumab), antibodies directed against programmed cell death protein-1 (PD-1, e.g., nivolumab, pembrolizumab, and cemiplimab), and anti-PD-1 ligand (PD-L1, e.g., atezolizumab, durvalumab, and avelumab) [1]. These drugs can be used alone or in combination with other immunotherapy [2] or chemotherapy [3] to improve the survival of cancer patients. Currently, it has been used for the treatment of tumors of lung, kidney, liver, bladder and breast cancer, melanoma, and lymphomas [4–6]. ICIs improve the prognosis and quality of life of patients, whereas the increase of use also brings various immune-related adverse events (irAEs). The dermatologic, gastrointestinal, pulmonary, hepatic, and endocrine systems were most frequently involved.

Cases of neurological irAEs are rare, accounting for less than 3% [7]. So far, the best-characterized central nervous system irAEs are encephalitis and meningitis. And neurologic irAEs known to be most relevant to the peripheral nervous system are peripheral neuropathies, GBS, myasthenia gravis, and myositis [8]. Once they occur, such as encephalitis, Guillain-Barré syndrome, or myasthenia gravis, they can develop into serious consequences or even death.

Epidemiology shows that nearly two-thirds of patients with GBS have a recent history of infection before the illness [9]. GBS with potentially life-threatening consequences occurs in approximately 0.1-0.2% of patients treated with ICIs [10]. To date, information on the incidence, characteristics, and outcomes of GBS associated to ICIs treatment is very limited. And the available information varies widely in diagnosis and treatment. Multidisciplinary treatment of tumors urgently requires neurologists and oncologists to accurately understand the clinical manifestations and treatment of ICI-related GBS.

This review summarized the published data on GBS or GBS-like disease occurring in patients after treatment with ICIs from 1990 up to date and analyzed their time patterns of occurrence, clinical presentation, diagnosis, treatment, and prognosis.

## 2. Methods

A systematic literature search was performed in PubMed and Embase for all case reports of GBS associated with ICIs published in English reporting on human beings from 1990 up to date. For the case reports search, the keywords used were as follows: [“Guillain-Barré Syndrome” OR “acute inflammatory demyelinating polyradiculoneuropathy” OR “Miller Fisher Syndrome” OR “acute motor axonal neuropathy” OR “acute motor-sensory axonal neuropathy”] AND [“Immune Checkpoint Inhibitors” OR “Immune Checkpoint Blockers” OR “PD-L1 Inhibitors” OR “Programmed Death-Ligand 1 Inhibitors” OR “CTLA-4 Inhibitors” OR “Cytotoxic T-Lymphocyte-Associated Protein 4 Inhibitors” OR “PD-1 Inhibitors” OR “Programmed Cell Death Protein 1 Inhibitor”]. Abstracts of medical conference were excluded. For each case, we extracted data on demographics and clinical manifestations and adjuvant examinations (imaging, cerebrospinal fluid, and electrophysiology). If gender, age, GBS clinical variation [11], electrophysiological subtype [12], or results of the relevant examination were not explicitly reported in the article, this case could not be considered for analysis. The search was conducted by Yan Li and Xiuchun Zhang. The selection of the articles should be agreed upon by the above two persons.

GraphPad Prism 8 was used for statistical analysis, and continuous data were expressed in the form of mean ± standard deviation or median. *P* < 0.05 was considered statistically significant. Since the proportion of males and females in the included cases was significantly different, unpaired *t*-test was used to analyze the ages of males and females.

Because the patient's personal information was provided in the original case report, authorization from the Ethics Committee was not required for this study.

## 3. Results

Using the search terminology, 38 case reports were identified from our database search, covering the period from January 2008 to February 2021. Three patients were excluded due to lack of age and CSF protein concentration, respectively. One case report was excluded because it was written in Japanese. Two case reports were not included because the diagnosis of GBS was ambiguous due to a disease progression similar to acute-onset CIDP. In addition, two patients with a history of GBS had no serious toxicities or deterioration of the previous autoimmune disorders after ICI therapy. The two patients were also excluded. A total of 30 case reports (total patients = 33) were used for final analysis [13–42]. According to the diagnostic criteria of GBS in NINDS, all the above 33 cases were consistent with the features of GBS. The clinical data and diagnostic details of all included patients were summarized in Tables 1–3.

### 3.1. Demographic Characteristics

GBS cases (*n* = 33) were from the United State (*n* = 13), United Kingdom (*n* = 3), Japan (*n* = 2), Italy (*n* = 2), Belgium (*n* = 2), Australia (*n* = 2), China (*n* = 1), Greece (*n* = 1), Netherlands (*n* = 4), France (*n* = 2), and German (*n* = 1) (Table 1). Of the 33 cases, twenty-six of these cases were male and seven were female, with an average of 62.2 ± 11.1 years (median: 65 years and range: 37-81 years). There was a male preponderance in 33 ICI-associated GBS patients we collected, with 3.7 times as many cases as female (26 vs. 7 cases: 78.8% vs. 21.2%). There was a significant difference between male and female ages at onset (mean: 64.4 ± 10.3 vs. 54.1 ± 11.1 years, *P* = 0.0278). The reports of comorbidities were variable, and no epidemics of specific diseases had been observed, so we did not analyze the comorbidities.

### 3.2. Immune Checkpoint Inhibitors and Tumor Type

Of ICI-associated GBS patients, sixteen patients exposed to nivolumab, eleven patients were investigated with ipilimumab, and seven patients were treated with pembrolizumab. Of these, four patients received nivolumab in combination with ipilimumab, and one patient received nivolumab in combination with pembrolizumab. In the above cases, the types of tumor the patients suffered from were melanoma (*n* = 20) [13, 14, 16, 17, 23, 24, 28, 29, 31–37, 39–42], lung tumor (*n* = 7) [18, 21, 25, 26, 28, 30, 38], urinary tumor (*n* = 5) [13, 15, 19, 20, 22], and nasal cancer (*n* = 1) [27]. The details were summarized in Tables 4 and 5.

### 3.3. Clinical Features of GBS Spectrum

The onset time of symptoms in 32 patients was analyzed, and the results suggested that the median time was 8.2 weeks after the initiation of ICI treatments. The minimum was 0.7 weeks (5 days) [37], and the maximum was 59 weeks [19]. As shown in Table 1, the time to neurological plateau was mentioned in 23 cases, with an average of 16.0 ± 8.4 days (range 7-44 days). The most common manifestations of GBS associated with ICIs were weakness (93.9%, 31/33), hyporeflexia or areflexia (90.9%, 30/33), and paresthesia (81.8%, 27/33). Among them, sensory symptoms and paraparesis or tetraparesis cooccurred in 45.5% (15/33) of cases. Other less common symptoms included cranial nerve involvement (20.7%, 7/33), dysphagia or dysarthria (18.2%, 6/33), respiratory symptoms (21.2%, 6/33), and ataxia (9.1%, 3/33). There were also 2 rare cases of GBS patients with dysautonomia as the onset symptoms [17, 36]. The Hughes Functional Grading Scale (HFGS) [43] was used to evaluate the clinical severity, and higher numbers indicated more severe disability. The average was 3.85 ± 1.42, with a high score of 6 and a low score of 1 (Table 2).

### 3.4. Electrophysiological, CSF, and Imaging Results

Twenty-seven patients underwent electrophysiological examination, and 18 cases had detailed electrophysiological reports. In the remaining cases, only the GBS subtype was mentioned. 54.5% (18/33) of cases were consistent with AIDP [16–19, 21, 22, 24, 26–30, 33, 34, 39, 42] and axonal damage accounted for 12.1% (4/33, AMSAN: 3 and AMAN: 1) [15, 23, 25, 27]. The subtypes in four cases were equivocal because they showed mixed features of axonal injury and demyelination [23, 31, 35, 36]. There were also three cases, two with MFS [13, 20] and the other with MFS-GBS overlap [32]. Regrettably, there were two cases of GBS diagnosis with no mention of subtypes [13, 38, 40].

CSF analysis was performed in 31 out of the 33 cases. The average protein level of CSF in patients with GBS related to ICIs was 180.68 ± 152.51 mg/dl (range: 37-680 mg/dl). 63.6% of the cases (21/33) presented with typical albuminocytological dissociation (cells ≤ 5/*μ*l and CSF protein > 45 mg/dl) [14, 16, 20–22, 24, 28, 30–34, 38, 40–42]. The median value of CSF protein was 124.5 mg/dl (range: 73-680 mg/dl). Slight pleocytosis (i.e., cell count > 5/*μ*l) was detected in 4/33 cases (12.1%) with a maximum cell count of 15/*μ*l and a median CSF protein of 115 mg/dl [15, 19, 27, 37]. CSF protein values and cell counts were normal in three cases [23, 25, 35], and there was elevated CSF immunoglobulin G levels in one case [25].

MRI was performed in 54.5% (18/33) of cases, of which both brain and spinal cord were examined in 8 cases and only spinal cord was 10 cases. MRI results showed nerve root involvement in 4 cases [16, 27, 29, 31], severe spinal stenosis in 1 case [19], cranial nerve involvement in 1 case, and normal or no metastatic signs in the rest [29].

### 3.5. Treatment and Prognosis of GBS

Twenty-seven cases (81.8%) were treated with intravenous immunoglobulin (IVIG), sixteen of the 27 patients with a combination of steroid therapy and one with PLEX. Seven of the 27 patients were treated with all three treatments. Five cases were treated with steroid therapy alone, and one case was treated with steroid combined with PLEX. No patients were treated with PLEX alone.

After treatment, 24 cases (72.7%) recovered or had only mild residual dysfunction, with two of them rechallenged ipilimumab [14] or pembrolizumab [21], and 5 cases had residual mild paresthesia [13, 14, 26, 29, 33]. Among the 33 ICIS-related GBS patients, 5 patients developed tumor progression after discontinuation of ICIS treatment, including 4 patients with melanoma [14, 31, 32, 37] and 1 patient with bladder cancer [22]. Of the eight deaths, three died of the respiratory muscle paralysis caused by GBS [17, 38, 41], 2 died of infection [13, 39], and three were due to progression of the tumor caused by ICI discontinuation [16, 28, 40].

## 4. Discussion

The effects of checkpoint inhibition affect a wide range of system and trigger a wide range of autoimmune toxicity. The exact mechanism of neurological irAEs in ICI-treated patients is unknown [44]. The existence of shared antigens between the tumor and itself may be one possible mechanism, such as gangliosides found in both melanoma and Schwann cells [45]. ICI-related GBS is rare as a type of neurological irAEs, but it can develop into life-threatening consequence once occurred. Diagnosis of ICI-related GBS should be made in the shortest possible time so as not to delay the administration of immune-regulation therapy.

There was a male preponderance in 33 ICI-related GBS patients we collected, with 3.71 times as many cases as female (male 26 and female 7). However, among GBS triggered by infection, males were affected only 1.5 times more frequently than females [11]. An epidemiological study on the risk factors of melanoma showed that the incidence of melanoma of men was almost three times that of by women by the age of 75 [12]. The incidence of lung tumor in men has historically been higher than in women, although the incidence of lung tumor in women has risen since the 1960s, especially in younger women [46–48]. Perhaps the higher incidence of cancer in men, resulting in more opportunities for men to use ICIs, was one of the reasons for the higher incidence of ICI-related GBS. Besides, ICIs were more effective for male cancer patients than female patients [49]. And the development of irAEs was related to the beneficial effects of immunotherapy in malignant tumors, especially in advanced-stage melanoma, advanced and metastatic NSCLC, and advanced renal cell carcinoma (RCC) [50]. The high effectiveness of ICI treatment in male patients may be also responsible for the male preponderance in ICI-related GBS.

In this study, melanoma had the largest number of tumor type that caused GBS after ICI treatment. Neurological irAEs were rare, with an incidence of less than 3% [8], whereas the overall incidence of severe nerve injury in melanoma patients treated with nivolumab with or without ipilimumab reached 0.93% [7], nearly one-third of the total neurological irAEs. Well know, GBS is a multifactorial autoimmune disorder, cell-mediated immunity plays an important role in immunopathology of all types of GBS. The activation of T cell caused by bacterial and virus leads to the production of cytokines and the release of free radicals, thus resulting in segmental demyelination [51]. And the cross-reaction between B-cell autoantibodies and axon gangliosides leads to axonal degradation [51]. Melanocytes and Schwann cells originate from the neural crest and have many common epitopes in humoral and cellular immune responses [52]. T cells regulate the degree of initial response of T cells by upregulating CTLA-4, while PD-1 inhibits the response of T cells in peripheral tissues and plays an important role in immune self-tolerance [53]. Due to the cross-reaction of molecular mimicry, T cell-mediated autoimmunity against melanoma cell antigens may also have an effect on the myelin antigens on Schwann cell membranes. In addition, the response rate of melanoma to ICIs was higher than other tumors [54]. A FAERS database-based clinical study also showed that patients with melanoma or non-small-cell lung cancer maybe at higher risk of fatal neurologic AEs [55]. Therefore, the author speculated that melanoma patients were more likely to develop GBS after ICI treatment than other types of tumors.

Existing reports suggested that the overall incidence of neurological adverse events (nAEs) at all levels was 3.8% for anti-CTLA-4 and 6.1% for anti-PD-1/PD-L1 [56]. CTLA-4 and its ligands are only expressed on immune cells, while PD-1 and PD-L1 (ligands of PD-1) are expressed on both immune and nonimmune cells in peripheral tissues [57]. The difference in spatial distribution between the CTLA-4 and PD-1 pathways may explain the high incidence of GBS in patients treated with nivolumab.

Many literatures had reported the timing of irAEs onset after the initiation of ICI treatments. The skin manifestations appeared at 2-3 weeks, and immune-mediated colitis, hepatitis, pneumonitis, and nephritis appeared approximately 5-10 weeks, 12-16 weeks, 8-14 weeks, and 14-42 weeks, respectively. Endocrine dysfunctions appeared from 9 weeks [58]. At present, there is no literature on the time to onset of neurologic symptom related to GBS caused by ICIs. The initial time of symptoms in 33 patients in this article was analyzed, and the results suggested that the median time was 8.2 weeks after the initiation of ICI treatments. Compared with GBS caused by infectious triggers, neurologic symptom appeared much later. This conclusion was expected to be helpful for the rapid diagnosis of ICIs-GBS.

Of the 33 GBS related to ICI patients we collected, the initial symptoms were very similar to infection-triggered GBS. Lumbar puncture was performed, and CSF analysis revealed elevated protein levels and albuminocytologic dissociation. A study of 962 patients with infection-induced GBS showed that the average protein level was 113.8 ± 11.8 mg/dl (range: 18–450 mg/dl) [59]. In this study, the average protein level of CSF in patients with GBS related to ICIs was 180.68 ± 152.51 mg/dl (range: 37-680 mg/dl). From a numerical point of view, the levels of CSF protein level of ICI-related GBS seemed to be higher than those of GBS induced by infection. Although it was not possible to distinguish infection-induced GBS from ICI-induced GBS based on CSF protein levels, lumbar puncture and protein-cell-separation were valuable in differentiating a variety of diseases such as spinal cord compression, metabolic diseases, side effects of drugs, vasculitis, and chronic inflammatory demyelinating polyneuropathy. In addition to elevated protein levels, lymphocytosis may also occur [37]. A similar pattern was observed in this study, with 4 patients showing a mild lymphocytosis in the CSF. However, multiple cases had been reported of lymphocytosis in the CSF of patients with infection-induced GBS [60–63]. Obviously, lymphocytosis in the CSF was not a characteristic of ICI-related GBS.

The electrophysiological results detailed in this study indicated that ICI-related GBS was a generalized, sensorimotor polyneuropathy characterized by mixed axonal/demyelination. An electrophysiological study of ICI-related peripheral neuropathy showed that immune-mediated neuropathy mainly manifested as demyelination of motor nerves, followed by length dependent axonal loss in sensory nerve [60]. The results of this study were in part consistent with the above conclusions. No matter what kind of electrophysiological changes were closely related to the autoimmune response of ICIs against peripheral nerve tissue, the onset of symptoms of neuropathy was closely related to ICI treatment, and corticosteroid or immune-regulatory therapy had good results.

In the treatment of all 33 patients, the immediate discontinuation of ICIs was an uncontroversial decision. Most patients received IVIG after discontinuation, with 16 patients receiving both IVIG and steroid therapy. Despite steroids were generally not recommended for treatment in infection-induced GBS, in ICI-related GBS, both American Society of Clinical Oncology (ASCO) Clinical Practice Guideline and National Comprehensive Cancer Network (NCCN) guidelines stated that trial of (methyl) prednisolone 1-2 mg/Kg was reasonable [64, 65], especially when CSF pleocytosis was higher than being anticipated for GBS [10]. And if the symptoms deteriorated, plasma exchange or IVIG treatment could be considered [66].

Among the 33 ICI-related GBS patients, 5 patients developed tumor progression after discontinuation of ICI treatment. One of the four patients with progressed melanoma rechallenged different class of immunotherapy, with significant and sustained response nearly 1 year later and no recurrence of GBS-like neuropathy. Whether or not to retreat with ICIs and whether to retreat with the same or different ICIs are challenges for oncologists. There is also limited data on the clinical efficacy and safety of retreatment. The current guidelines suggested that corticoid therapy and temporary or permanent discontinuing ICIs were required for grade ≥ 2 irAEs and permanently discontinuing ICI treatment for grade 4 irAEs [65, 67]. A study had shown that the recurrence rate of the same irAEs resulting in discontinuation of ICI treatment in cancer patients who rechallenged the same ICI was 28.6% (anti-PD-1 or anti-PD-L1 monotherapy), 47.4% (anti-CTLA-4 monotherapy), and 43.5% (combination therapy), respectively [66]. The recurrence rate of irAEs varied depending on the organ involved in the initial irAEs, with gastrointestinal irAEs having the highest recurrence rate [68]. And the variables associated with a higher recurrence rate of irAEs were anti-CTLA-4 regimen, age, colitis, hepatitis, and pneumonia, in order [68]. In addition, the duration from ICI discontinuation to rechallenge, and the severity of the initial irAEs did not predict whether irAEs would reappear after rechallenge of ICIs [69]. However, no relevant literature has been reported on whether discontinuation due to ICI-related GBS can rechallenge the same or different ICIs again.

In addition, paraneoplastic peripheral neuropathy should be excluded in the diagnosis of ICI-related GBS. Paraneoplastic peripheral neuropathy is a remote effect of the malignancy mediated by the immune system. It develops prior or during a cancer and is independent of tumor infiltration or cancer therapy [70]. Paraneoplastic sensory neuropathy is the most frequent in this group of disorders, and motor, autonomic, or central nervous systems are also involved [71]. Date on the treatment of paraneoplastic peripheral neuropathy is limited, and the combination of malignancy therapy with immunomodulatory therapies such as corticosteroids, IV immunoglobulin, or immunosuppressants may be effective [72]. However, ICI-related GBS occurred in malignant tumors after ICI treatment, and the immunomodulatory therapeutic effect was obvious. This is the most obvious difference between the two.

Here are also several limitations in this study. First, the number of the included cases was small which limited us to perform subgroup analysis. Second, patients with various cancers were included, which might have bias in the incidence of some adverse effects.

## 5. Conclusion

Elderly male patients with melanoma were most likely to develop ICI-related GBS. And the median duration was 8.2 weeks after the initial ICI treatment. In order to make a final diagnosis, physicians need to collect specific neurological symptoms and signs and combine them with CSF analysis and electrophysiological examination. In addition, imaging is required to exclude tumor metastasis. Immediate termination of ICIs followed by IVIG in combination with high-dose steroids therapy or PLEX and supportive treatment could lead to a better prognosis.

## Figures and Tables

**Table 1:** Main characteristics and diagnostic details of included cases.

Case	Article	Country	Age	Sex	Tumor type	ICIs	Time of GBS onset after the initiation treatment (week)	Time to neurological plateau (days)	GBS diagnosis
1	Muralikrishnan, S et al. [14]	USA	65	Female	Melanoma	Pembrolizumab	4 weeks	44	Clinical+CSF+electrophysiology
2	Han, C et al. [15]	China	55	Male	RCC	Pembrolizumab	16 weeks	No mention	Clinical+CSF+electrophysiology
3	Arora, A et al. [16]	USA	70	Male	Melanoma and prostate cancer	Pembrolizumab	12 weeks	16	Clinical+CSF+electrophysiology
4	Yuen, C et al. [17]	USA	66	Male	Melanoma	Nivolumab	12 days (1.7 weeks)	34	Clinical+CSF+electrophysiology
5	Pomerantz, M et al. [18]	Italy	58	Male	SCLC	A combination of nivolumab and pembrolizumab	59 days (8.4 weeks)	No mention	Clinical+CSF+electrophysiology
6	Pierrard, J et al. [19]	Belgium	70	Male	Urothelial carcinoma	Nivolumab	59 weeks	14	Clinical+CSF+electrophysiology
7	McNeill, C. J et al. [20]	UK	68	Male	RCC	Nivolumab	8 weeks	13	Clinical+CSF+electrophysiology
8	Kyriazoglou, A et al. [22]	Greece	74	Male	Bladder cancer	Nivolumab	8 weeks	15	Clinical+CSF+electrophysiology
9	Mazzaschi, G et al. [21]	Italy	80	Male	Lung adenocarcinoma	Nivolumab	12 days (1.7 weeks)	22	Clinical+CSF+electrophysiology
10	Gravbrot, N et al. [23]	USA	71	Male	Melanoma	Ipilimumab	10 weeks	Several days	Clinical+CSF+electrophysiology
11	Wilson, R et al. [24]	UK	52	Male	Melanoma	Nivolumab Ipilimumab	4 weeks	7	Clinical+CSF+electrophysiology
12	Thapa, B et al. [25]	USA	60	Male	Lung adenocarcinoma	Nivolumab	14 weeks	No mention	Clinical+CSF+electrophysiology
13	Ong, S et al. [26]	UK	66	Male	Lung adenocarcinoma	Pembrolizumab	6.5 weeks	16	Clinical+electrophysiology
14	Nukui, T et al. [27]	Japan	45	Male	Nasal cancer	Nivolumab	10 weeks	14	Clinical+CSF+electrophysiology
15	Manam, R et al. [28]	USA	73	Male	Lung adenocarcinoma	Pembrolizumab	3 weeks	No mention	Clinical+CSF
16	Manam, R et al. [28]	USA	81	Male	Melanoma	Pembrolizumab	10 weeks	No mention	Clinical+CSF+electrophysiology
17	Garcia, C. A et al. [29]	USA	55	Male	Melanoma	Ipilimumab	6 weeks	No mention	Clinical+CSF+electrophysiology
18	Fukumoto, Y et al. [30]	Japan	66	Male	NSCLC	Nivolumab	3 weeks	16	Clinical+CSF+electrophysiology
19	Cafuir, L et al. [31]	USA	42	Male	Melanoma	Ipilimumab	10 weeks	13	Clinical + CSF + electrophysiology
20	Baird-Gunning, J. J. D et al. [31]	Australia	58	Female	Melanoma	Nivolumab Ipilimumab	10 days (1.4 weeks)	12	Clinical+CSF+electrophysiology
21	Supakornnumporn, S et al. [33]	USA	77	Male	Melanoma	Nivolumab Ipilimumab	10 weeks	14	Clinical+CSF+electrophysiology
22	Schneiderbauer, R et al. [34]	German	51	Male	Melanoma	Nivolumab	20 weeks	No mention	Clinical+CSF+electrophysiology
23	Patel, R. J et al. [35]	USA	71	Male	Melanoma	Ipilimumab	10 weeks	10	Clinical+electrophysiology
24	Kelly Wu, W et al. [36]	USA	37	Female	Melanoma and papillary thyroid cancer	Ipilimumab	No mention	9	Clinical+CSF+electrophysiology
25	Gu, Y et al. [37]	Australia	49	Female	Melanoma	Ipilimumab Nivolumab	5 days (0.7 weeks)	11	Clinical+CSF+electrophysiology
26	Jacob, A et al. [38]	USA	68	Female	Lung squamous and cell carcinoma	Nivolumab	12 weeks	14	Clinical+CSF+electrophysiology
27	Gaudy-Marqueste, C et al. [40]	France	65	Male	Melanoma	Ipilimumab	6 weeks	11	Clinical+CSF
28	Bot, I et al. [41]	Netherlands	63	Male	Melanoma	Ipilimumab	12 weeks	A few days	Clinical+CSF+electrophysiology
29	Wilgenhof, S et al. [42]	Belgium	57	Female	Melanoma	Ipilimumab	8 weeks	No mention	Clinical + CSF + electrophysiology
30	de Maleissye, M.F. et al. [39]	France	45	Female	Melanoma	Pembrolizumab	9 weeks	21	Clinical+CSF+electrophysiology
31	Janssen, J.B.E. et al. [42]	Netherlands	74	Male	Prostate cancer	Pembrolizumab	6 weeks	21	Clinical+CSF+electrophysiology
32	Janssen, J.B.E. et al. [13]	Netherlands	67	Male	Melanoma	Nivolumab	3 weeks	7	Clinical+CSF
33	Janssen, J.B.E. et al. [13]	Netherlands	55	Male	Melanoma	Pembrolizumab	16 weeks	15	Clinical+CSF

LEs: lower extremities; UEs: upper extremities; NSCLC: non-small-cell lung cancer; SCLC: small-cell lung cancer; RCC: renal cell carcinoma. Time to Nadir: days between the onset of neurological symptoms and the development of the worst clinical symptoms (no progression).

**Table 2:** The clinical data and diagnostic details of all included patients.

Case	GBS clinical picture						Hughes scale
	Onset	Motor	Sensory	Reflex	Autonomic disturbances	Respiratory involvement	
1	LEs ascending numbness bilaterally	Muscle weakness in lower limbs and left hand	Left tongue numbness and diminished facial sensation.	Generalized areflexia	None	No	2
2	Weakness and numbness of the extremities	A mild decrease in muscle strength of the limbs	Paresthesia of the four extremities	Absence of tendon reflex of the limbs. No pathological reflex.	None	No	1
3	Progressive bilateral LE weakness	Decreased strength in lower extremities	Slight impairment of touch and pinprick sensation, worsening back pain and painful burning in feet bilaterally	Bilaterally absent patellar and ankle reflexes	None	No	6
4	Bilateral finger and toe paresthesia followed by weakness of the lower and then upper limbs		Absent pinprick sensation and vibratory sensation	Generalized areflexia	Tachycardia hypotension	Respiratory distress	6
5	Bilateral LE paresthesias, pain	Bilateral LE weakness and progressively worsened to a complete inability to ambulate	Diminished sensory perception in LEs, pinprick over all distal extremities, marked decrease in vibration sensation and absent proprioception in LEs	Generalized areflexia	None	No	4
6	Rapidly progressing weakness of LEs	Rapidly progressing upper limb weakness over 2 weeks	None	Normal	None	No	3
7	Progressive weakness and sensory disturbance in face and limbs	General weakness. slurred speech, double vision, difficulty swallowing, within 72 hours, rapidly worsening bulbar symptoms	Paresthesia of his hands and feet, an unsteady gait	Normal tone, power and deep tendon reflexes	None	Shortness of breath	5
8	Muscle weakness and fatigue	Severe proximodistal weakness in the UEs and LEs	Impairment of position sense, vibration, stereognosisn, and graphesthesia	Areflexia of the 4 limbs and absent pathologic reflexes	None	No	3
9	Paresthesia and burning pain arose in LEs, followed by a progressive bilateral weakness	Symmetric distal dominant weakness and dramatically worsened. Loss of fine motor control on distal UEs	Pain and sever numbness.	Hypoareflexia and decreased deep tendon reflexes	None	No	4
10	Severe, progressive, symmetric ascending weakness	The paralysis progressed to inability to stand and arm weakness Mild dysphagia	Without sensory loss	Unobtainable deep tendon reflexes	None	Shortness of breath	4
11	Headache and generalized tiredness	Bilateral facial weakness, distal limb weakness	Progressive hand and feet numbness	Reduced deep tendon reflexes	None	No	3
12	Severe weakness of all extremities	Mild to moderate decrease in motor strength in all 4 extremities, LEs worse than UEs	Sensory loss of vibration/proprioception in bilateral LEs	Diminished reflexes in bilateral LEs but normal reflexes in bilateral UEs	None	No	4
13	LE pain, paresthesia and weakness	UE weakness and a lower motor neuron pattern of right-sided facial weakness Bilateral, predominantly distal LE weakness	Reduced sensation	Generalized areflexia	None	No	2
14	Diplopia, muscle weakness, and numbness of extremities	Bed-bound Right external ophthalmoplegia, left facial nerve palsy and bulbar palsy, severe muscle weakness	Bilateral facial dysesthesia	Absent deep-tendon reflexes	None	No	4
15	Generalized weakness	Progressive weakness in the bilateral LEs greater than the UEs	Without sensory loss	Absent deep tendon reflexes in the bilateral UEs and LEs	None	No	3
16	Progressive weakness in the bilateral LEs and then spreading to the bilateral UEs	A strength of 2/5 in the bilateral UEs and 0/5 in the bilateral LEs and no bulbar muscle weakness	Without sensory loss	Areflexia	None	Requiring mechanical ventilation	4
17	Paresthesias in distal LEs bilaterally	Ascending weakness	Ascending paresthesias	Loss of deep tendon reflexes	None	No	2
18	Muscle weakness of the LEs	Weakness rapidly progressed and became bed-bound	Paresthesias of the distal limbs	Absence of deep tendon reflexes of the four extremities	None	No	4
19	Bilateral thigh weakness and paresthesia of the soles of his feet	LEs weakness, worsened rapidly	Pain and numbness. Vibration in the toes impaired	Tendon reflexes in the LEs were absent.	None	No	3
20	Bilateral ptosis and external ophthalmoplegia	Proximal upper and lower limb weakness, neck flexion weakness	Marked truncal and limb ataxia were evident accompanied by impaired proprioception.	Areflexia	None	No	2
21	Rapidly progressive numbness and tingling sensation in both hands and feet, generalized muscle weakness with multiple falls	Severe distal and proximal muscle weakness in both upper and lower extremities	Loss of all sensory modalities in distal upper and lower extremities	Generalized areflexia	None	No	4
22	Muscular weakness in both legs and peripheral paresthesias	Muscular weakness in both legs	Formication in both hands, bilateral hypoesthesia of the legs up to the upper thigh	Absent tendon reflexes in both legs	None	No	1
23	Progressive LE weakness	Progressive and ascending muscle weakness	Without sensory loss	Absent deep tendon reflexes	None	No	4
24	Tonically dilated pupil, gastrointestinal dysmotility, urinary retention, and profound orthostatic hypotension	Worsening dysarthria, generalized weakness	Ascending paresthesias, absent proprioception	Absent deep tendon reflexes	None	No	5
25	Painful paresthesia in the extremities	Proximal loss of antigravity power and loss of independent mobility	Sensory loss	Absent reflexes	Nausea, postural hypotension constipation	No	4
26	Fatigue and bilateral LE weakness	Profound weakness in lower extremities, progressive loss of motor function	Tingling sensation in feet, progressive loss of sensory function	Loss of deep tendon reflexes in all extremities associated with complete lack of strength	None	Respiratory muscle paralysis	5
27	Pruritus, abdominal meteorism, and natisea	Symmetrical weakness in the 4 limbs	Mild bilateral hypoesthesia in the extremities	Disappearance of deep tendon reflexes	Abdominal meteorism and natisea	Worsening of the respiratory function	6
28	Paresthesias of the feet and fingertips and unsteady gait	A mild tetraparesis	Sensory loss in both hands and feet	Generalized areflexia	None	No	5
29	Dysesthesia (numbness and tingling) at the hands and feet	Rapidly ascending loss of motor function	Rapidly ascending loss of sensory function	Loss of the deep tendon reflexes	None	Respiratory insufficiency	5
30	Paresthesia and hypoesthesia of all limbs	Rapidly followed by symmetrical motor weakness in legs Peripheral facial paralysis	Paresthesia and hypoesthesia	Areflexia in the legs	None	No	6
31	Painful fingers and loss of taste	Weakness in all extremities	Sensory loss in all extremities	Areflexia in both legs	None	Respiratory function was affected	6
32	Muscular pains in his arms and legs without paralysis	Loss of motor functions in the hands and LEs	Loss of sensor functions in the hands and LEs	Areflexia of LEs	None	The respiratory function preserved	4
33	Ascending weakness, paresthesia, and sensory loss progressive	Weakness of the legs	Sensory disturbances of the lower extremities	Areflexia	None	The respiratory function preserved	3

LEs: lower extremities; UEs: upper extremities.

**Table 3:** Electrophysiology, CSF, imaging, treatment, and clinical outcomes.

Case	NCS finding	CSF		Imaging	Treatment	Outcome
		Protein (15-45 mg/dl)	Cell (<5/*μ*l)			
1	4 weeks after ICIs: not suggestive of neuropathy	78 OL (+)	17		IVIG over 5 days for the first time	Significant improvement in muscle weakness. 18 days after discharge, the disease developed an ascending paralysis of the extremities.
	44 days later: acute and chronic demyelinating polyneuropathy	175 OL (-)	3		PLEX for 7 days and solumedrol for 5 days instead of IVIG for reoccurrence	Melanoma progression occurred about 1 year after the last dose of pembrolizumab. Rechallenging with ipilimumab. Only ongoing bilateral leg tingling and diarrhea
2	Acute motor and sensory axonal neuropathy	58.33	8	MRI of the brain and spinal cord was normal.	Oral treatment with dexamethasone and then switched to IVIG along with prednisone	Symptoms improved during 1 week of treatment. After 1 month, walking and standing was normal. No recurrence of tumor
3	AIDP	405	4	Spinal MRI: abnormal thickening and enhanced posterior nerve roots (L4-L5, L5-S1), no features of metastatic disease	Dexamethasone for 6 days and IVIG for 5 days	Hydrocephalus, ventricular enlargement and bed-bound Died after withdraw from life support
4	Dysautonomia secondary to AIDP	91	0		IVIG+prednisone+PLEX Life-sustaining therapies	Respiratory distress, tachycardia, and hypotension Died after withdrew life-sustain
5	AIDP	124	41	Brain as well as the lumbar and thoracic spine MRI: no clear cord or cauda equina involvement despite spine metastases	IVIG for 5 days	Rehabilitation No other potential agents were given and alive 6 months after the use of last immunotherapies.
6	AIDP	105	8	Spine MRI: severe stenosis of lumbar spinal canal	IVIG for 5 days and methylprednisolone during 7 days	A rapid clinical improvement was observed within the first 3 days. Methylprednisone was tapered progressively over 10 weeks. Completely recovered. 6 months later, cancer remains stable.
7	MFS	175	5	Brain and cervical spine MRI: normal	IVIG and intravenous methylprednisone followed by oral prednisolone	Severe global limb weakness and respiratory decompensation developed after 3 days of IVIG. Respiratory function and motor weakness improved after 9 days of corticosteroid treatment. Rehabilitation after 3 months of treatment
8	AIDP	140	2	Brain and cord MRI: nonspecific cerebral white-matter lesions	IVIG for 5 days along with prednisone for 1 month	Improvement. Disease progressed with increased number and size of lung metastases. Died from sepsis
9	AIDP	88	2	Brain and spinal MRI: no evidence of metastases or ischaemic and/or hemorrhagic lesions	Methylprednisolone IVIG for 5 days Prednisone	Without clinical benefit Leg weakness, numbness in patient's fingers, and paresthesia dramatically improved. A further improvement was obtained after 2 weeks of prednisolone.
10	Acute sensorimotor polyradiculoneuropathy with mixed axonal/demyelinating	<45	<5	Spinal CT: normal	IVIG for 5 days and prednisone 30 mg daily at the same time	3 weeks later, improved rapidly and transition to pembrolizumab with no evidence of GBS
11	AIDP	230	0	Spinal MRI: normal	IVIG	Good recovery
12	AMAN	37 mg/dl but elevated IgG levels	2		Prednisone IVIG	Symptoms continued to worsen. Minimally improved but prevented further progression
13	AIDP	No lumbar puncture was performed.		Spinal MRI: degenerative changes with no evidence of cord compression	Methylprednisone and IVIG Prednisone	19 days after admission, weakness and numbness mostly resolved. 1 month later, neurological recovery except for mild residual paresthesias of the feet
14	Multiple cranial neuropathy and AIDP	350	7	Lumbar spine MRI: a reduction of gadolinium enhancement of nerve roots and cauda equina	IVIG Steroid pulse therapy	Multiple cranial neuropathies were moderately improved after 4 weeks of treatment. Muscle weakness remarkably improved after the 3 courses of therapy.
15	AIDP	680	<5		Methylprednisolone along with IVIG PLEX	Strength diminished to 2/5 in the bilateral UEs and LEs. Respiratory status worsened. Respiratory status improved, and motor function gradually recovered.
16	AIDP	560	<5	Brain CT: a hemorrhage within one of his metastatic lesions and associated vasogenic edema	Methylprednisolone along with IVIG for 5 days PLEX	Acute hypoxic respiratory failure, requiring mechanical ventilation. After five days of treatment without any clinical improvement. A hemorrhage within one of metastatic lesions and associated vasogenic edema. Died after the withdrawn of care
17	AIDP	175	Lymphocytic pleocytosis	Brain and spinal MRI: abnormal enhancement involving the bilateral 5th, 7th and 8th cranial nerves, cauda equina nerve roots as well as the conus surface and peripheral nerves at the thoracolumbar junction	Methylprednisolone	Motor symptoms in hands and lower extremities improved rapidly after 2 days and gradually recovered over a 12-week period. Weakness resolved completely, residual minimal paresthesias
18	AIDP	339	4		Prednisone (60 mg/day) IVIG (0.4 g/kg) for 5 days	Symptoms worsened Gradually improved 3 months later, he was able to walk with a cane.
19	Asymmetric, subacute to early chronic and ongoing lumbar polyradiculoneuropathy with axonal involvement and demyelinating	>300	1	Spinal MRI: diffuse enhancement surrounding the entire conus and all of the nerve roots	Dexamethasone	Partial response systemically and neurologic improvement 6.5 months later, melanoma progressed.
20	MFS and demyelinating sensorimotor polyneuropathy	125	0		IVIG 2 g/kg for 5 days and methylprednisolone followed by a weaning dose of oral prednisolone PLEX	Modest clinical improvement Significant functional improvement and complete recovered at last. But melanoma had progressed.
21	AIDP	86	0		IVIG over 5 days Prednisone 90 mg/day	Dysphagia requiring nasogastric tube for feeding After 6 months, neurological condition improved significantly and the dysphagia completely was resolved. Numbness was still present but improved.
22	AIDP	73	<5		IVIG for 5 days Methylprednisolone	Did not lead to any clinical improvement Clinical recovery started 48 h later and was nearly complete after 6 weeks.
23	Acute sensorimotor polyradiculopathy with mixed axonal/demyelinating features	39	<5	Thoracic spine and lumbar spine CT: degenerative changes	IVIG	Marked improvement on treatment day 5
24	GBS presenting as dysautonomia, a length-dependent, sensorimotor polyneuropathy with axonal and demyelinating properties	No lumbar puncture was performed.			IVIG	Dysautonomia and weakness persisted, and cardiovascular and respiratory status improved by 2 months. Persistent urinary retention, oropharyngeal dysphagia, and generalized weakness
25	AMSAN with autonomic symptoms	115	15	Spinal MRI: normal	IVIG (0.4 g/kg/d) for 5 days Methylprednisolone (1 g/d for 5 days, then 500 mg/d for 3 days) followed by tapering oral prednisone (1 mg/kg/d) PLEX (5 changes over 2 weeks, followed by weekly exchanges)	Symptoms stabilized with mild improvement, yet one month later, it developed worsening weakness and ongoing painful paresthesia. Persistent nausea coupled with postural hypotensin and constipation 6 weeks later (12 weeks after initial treatment), the patient had only mild weakness. 9 months later, melanoma progressed.
26	GBS	85	0	Spine MRI: normal	IVIG+PLEX	Within 2 hours, respiratory muscle paralyzed, and ventilator support was applied. She was extubated after 11 days and expired within a few hours.
27	Acute, generalized, symmetrical, and sensorimotor neuropathy, impossible to distinguish axonal or demyelinating disorder because of severe limb edema	160	0		Methylprednisolone PLEX	Clinical status did not improve. Died of multivisceral failure within a few days
28	AMSAN	89	0	Cervical spine MRI: normal	IVIG	The muscle strength of all limbs slightly increased. But 3 days later, died from respiratory insufficiency
29	AIDP	167	<2	Brain and spinal MRI: normal	Methylprednisolone	Recovery
30	AIDP	56	Normal		Prednisolone IVIG	The neurologic symptoms reached the peak within 3 weeks and decreased over the next 2 months.
31	MFS	81 OL (+)	Normal	Central nervous system imaging showed no cerebral or vertebral pathology.	Prednisolone IVIG PLEX	A slight improvement with these treatments 2 months later, died from pneumonia
32	GBS	107.5	61		Prednisolone IVIG	The pain was greatly diminished. 8 months later, the motor and sensor function of extremities were still slowly recovering.
33	GBS	204.6 OL (+)	Normal		Prednisolone IVIG Methylprednisolone	The mild persistent weakness of his feet extensors and mild sensory loss and ataxia

AIDP: acute inflammatory demyelinating polyradiculoneuropathy; MFS: Miller Fisher syndrome; IVIG: intravenous immunoglobulin; PLEX: plasma exchange; AMAN: acute motor axonal neuropathy; AMSAN: acute motor and sensory axonal neuropathy; OL: oligoclonal bands.

**Table 4:** Tumor type and immune checkpoint inhibitors.

Tumor type		Monotherapy			Combination therapy	
		Pembrolizumab	Nivolumab	Ipilimumab	Nivolumab and ipilimumab	Pembrolizumab and nivolumab
Melanoma (20) (M : F = 14 : 6)		6	3	7	4	
Lung cancer (7) (M : F = 6 : 1)	NSCLS (1)		1			
	SCLS (1)					1
	Adenocarcinoma (4)	2	2			
	Squamous cell carcinoma (1)		1			
Urinary tumor (5) M: 5	RCC (2)	1	1			
	Urothelial carcinoma (1)		1			
	Bladder cancer (1) Prostate cancer (1)	1	1			
Nasal cancer (1) M: 1			1			

NSCLS: non-small-cell lung cancer; SCLS: small-cell lung cancer; RCC: renal cell carcinoma.

**Table 5:** Treatment and prognosis of ICIs-GBS.

Treatment (*n* = 33)	Progression		
	Recovery	Residual dysfunction	Death
IVIG (*n* = 3)	2		1
IVIG+steroid (*n* = 16)	8	6	2
IVIG**+**PLEX (*n* = 1)			1
IVIG+steroid+PLEX (*n* = 7)	2	2	3
Steroid (*n* = 5)	1	3	1
Steroid+PLEX (*n* = 1)			1
Total	13	11	9

IVIG: intravenous immunoglobulin; PLEX: plasma exchange.

## Abbreviations

ICIs: Immune checkpoint inhibitors
CTLA-4: Cytotoxic T lymphocyte-associated antigen-4
PD-1: Programmed cell death protein-1
PD-L1: Programmed cell death protein-1 ligand
irAEs: Immune-related adverse events
GBS: Guillain-Barré syndrome
AIDP: Acute inflammatory demyelinating polyneuropathy
AMAN: Acute motor axonal neuropathy
AMSAN: Acute motor and sensory axonal neuropathy
MFS: Miller fisher syndrome
DML: Distal motor latency
EMG: Needle electromyography
IVIG: Intravenous immunoglobulin
PLEX: Plasma exchange
CSF: Cerebrospinal fluid
CNS: Central nervous system
NSCLC: Non-small-cell lung cancer
RCC: Renal cell carcinoma.
